# Harnessing the healing power of the land: culturally appropriate treatments for Indigenous persons with a substance use disorder

**DOI:** 10.3389/fpsyt.2026.1796719

**Published:** 2026-03-23

**Authors:** Julie Wallace, Maryana Kravtsenyuk, Randal Bell

**Affiliations:** 1 Faculty of Medicine, Northern Ontario School of Medicine, Sudbury, ON, Canada; 2 Department of Psychiatry, University of Toronto, Toronto, ON, Canada; 3 Department of Psychiatry, University of Alberta, Edmonton, AB, Canada; 4 Wellness Division, Enoch Cree Nation, AB, Canada

**Keywords:** addiction, healing, Indigenous, land-based, substance use disorder, two-eyed

## Abstract

**Introduction:**

Substance Use Disorder (SUD) has become increasingly prevalent in Canada, disproportionately impacting Indigenous populations. Canada must do better; Indigenous peoples deserve better. In recent years, there has been growing recognition of the need to incorporate Indigenous culture into SUD treatment. Land-based healing strengthens connections to land and culture, which has been associated with improved mental health, self-esteem, and sense of identity, thereby promoting healing and recovery.

**Methods:**

An in-depth analysis of available literature surrounding land-based healing programs for SUD was conducted. Recent peer-reviewed scientific papers and journal articles were found through NOSM University library databases, PubMed, Science Direct, and Google Scholar. Grey literature, such as news articles and community websites, captured important first-person narratives and specific treatment center interventions. Emerging themes were used to develop recommendations for the future implementation of similar programs, with consultation with an Indigenous knowledge holder to ensure cultural integrity.

**Results:**

Literature revealed the healing power of land-based healing for Indigenous Persons with SUD. Keys to success included community involvement and governance, collaboration, holistic approaches to health, continuity of care, and the ability to reconnect with nature.

**Discussion:**

Challenges and recommendations in the future implementation of land-based healing programs for SUD include developing program evaluation and data-sharing methods that emphasize Indigenous knowledge translation and lived experiences, funding equity for Elders, and cultural safety training to promote collaboration and high-quality care. Through increasing awareness of land-based SUD treatments, more programs can be implemented across Canada that encourage culturally sensitive and effective healing options for Indigenous persons with SUD.

## 1 Introduction

In 2021, Keeseekoose First Nation in Saskatchewan declared a state of emergency for mental health and addictions after an increase in opioid overdoses (1). At that time, five children were orphaned yearly due to overdoses in Keeseekoose First Nation. This heartbreaking story is not unlike experiences faced by other Indigenous communities across Canada. In 2021, rates of hospital visits in Ontario for accidental drug poisonings were 7.2 per 10,000 for Indigenous populations, drastically higher than non-Indigenous populations, who experienced a rate of 0.8 per 10,000 (2).

Residential schools give way to the modern child welfare state, in which 53.8% of children in care are Indigenous (3). It is through these traumatic life events, and ongoing effects of colonization, that Indigenous children experience adverse childhood experiences, which increase the likelihood of adulthood addiction, chronic health conditions and early death (4, 5). Extreme poverty and social determinants of health in Indigenous communities exacerbate these problems, fueling the growing flames of SUD and the perpetuation of generational addiction cycles (5, 6).

There has been increasing recognition of the need to incorporate Indigenous knowledge into SUD treatments in Canada. Land-based SUD treatment focuses on rebuilding relationships with Mother Earth that may be strained due to trauma (7). It may strengthen connection to family, language, and culture while facilitating intergenerational knowledge sharing. Land-based treatment services often include hunting, fishing, medicine walks, building shelters, and harvesting, in conjunction with traditional ceremonies and teachings (2, 6–8, 13).

Elders and Knowledge Keepers facilitate land-based programs through guidance in the traditional aspects of healing (8). Land-based treatments work to heal the disconnect between Indigenous peoples and their culture, creator, and identity; thereby improving mental health, self-esteem, cultural identity, and communication skills (7, 8). Land-based healing is especially powerful for addiction treatment because it can support healthy lifestyles, positive coping mechanisms, and help heal underlying trauma.

The therapeutic impacts of nature have been demonstrated through the Three-Day Effect, in which a participant is completely immersed in nature for 72 hours (9). Through comparing outcomes before and after wilderness expeditions, the Three-Day Effect has been clinically shown to improve creativity, problem-solving, and cognitive function. Similarly, Forest Bathing is the Japanese practice of immersing oneself in nature (10). Studies show that Forest Bathing has positive therapeutic effects on depression, anxiety, cardiovascular, respiratory, and immune systems. Although the Three-Day Effect and Forest Bathing are not assessed in Indigenous populations, they provide evidence for the benefits of nature on healing from SUD (9, 10). These models differ from land-based healing due to the lack of additional cultural elements that are considered crucial in SUD. However, they provide supportive evidence of the health benefits of nature immersion that may be applied in land-based healing.

## 2 Methods

A narrative review was conducted through in-depth analysis of available literature on land-based healing for SUD, using online databases, including NOSM University Library, PubMed, Science Direct, and Google Scholar. Search terms included: Indigenous, land-based, substance use disorder, addiction, Two-Eyed, healing. The review included literature published between 2011 and 2026, with the inclusion of seminal foundational works when necessary and preference given to articles published within the last 10 years. Grey literature was used to capture first-person narratives.

Inclusion criteria included literature published within the last 15 years, programs based in Indigenous populations in Canada, and grey literature that provides first-hand perspectives. Exclusion criteria included literature focusing on land-based programs outside of Canada, publications from before 2011, and programs not intended for treatment of addiction or mental health.

The article screening process included an abstract review to assess relevance and inclusion criteria eligibility. Articles considered relevant were reviewed fully to determine if they aligned with the purpose of the narrative review. Emerging themes from literature were evaluated to develop recommendations for the future implementation of similar programs, with consultation with an Indigenous knowledge holder to ensure cultural integrity and alignment with traditional teachings.

## 3 Land-based healing approaches, successes, and challenges

### 3.1 Approaches to SUD healing rooted in Indigenous culture

Many land-based SUD programs incorporate the Two-Eyed Seeing approach, referring to the combination of Indigenous traditional knowledge and Western ways of thinking (8, 11). This is a valuable approach in SUD treatments for Indigenous persons as it respects the strengths of both Indigenous and Western viewpoints (12).

Interconnectedness of culture, land, and community is key in successful healing of SUD (13). To provide effective care for Indigenous persons experiencing addiction, services must be culturally supportive and create a safe space to affirm the healing process. Culturally supportive practices may include traditional teachings and ceremonies, like sweat lodge, fasting, smudging, sharing circles, Grandfather Teachings, and sacred song and dance (6, 13). Time spent with Elders in nature may facilitate intergenerational transmission of knowledge, specifically while learning about how to protect, value, and maintain the land (7). Through activities like hunting, gathering, medicine walks, and building shelters, a sense of identity may be strengthened, creating a positive mindset for overcoming addiction (8).

### 3.2 Keys to success in existing land-based treatments for SUD

Of existing land-based treatments for SUD, various keys to success have been identified. The *Thunderbird Partnership Foundation’s Substance Use Treatment and Land-Based Healing* report analyzed data from the *National Native Alcohol and Drug Abuse Program and National Youth Substance Abuse Program* treatment centers and found that community strength was a common theme in successful rehabilitation (7).

The Naandwe Miikan clinic in the Wiikwemikong Unceded Territory was initiated in response to addiction in the community (6, 14). Initially, Indigenous persons with SUD would travel far distances to clinics, making it difficult to access care. Eventually, they recruited healthcare providers and started their own culturally- based opioid agonist treatment clinic within the First Nation. Likewise, the Nishnawbe Aski First Nation NAN Hope Program is a community-based SUD program where referrals are not required and individuals can in-community access (7). In the NAN Hope Program, treatment retention rate after 18 months was 72%, significantly higher than the national average rate (7). This success was attributed to community-governance, on-site availability, and community accessibility of the program. Community-led treatment centers for SUD provide an opportunity to cater to specific needs, utilize local resources, appreciate lived experiences, and foster trust within the community (7, 8, 11).

When family and community become involved in healing, it may create support for the individual on their recovery journey (6, 8).

Continuity of care for Indigenous persons throughout SUD recovery may contribute to success (11). When participants leave SUD treatment, there is risk they may revert to unhealthy coping strategies. The Gwekwaadziwin Miikan Mental Health and Addiction Program is a three-phase, land-based program, including a three-month immersive nature learning program, followed by one year in supervised transitional housing, finishing with gradual reintegration into the community (15). The success of this program lies within the staging system that allows individuals to graduate to the next step of recovery while maintaining continuity of care.

Many Indigenous SUD treatment programs attribute their successes to the time spent with Mother Nature and the strengthened connection with culture, identity, language, and ancestors, promoting a healing mindset (2). For instance, the Nimkee NupiGawagan Healing Centre provides an equine program, allowing youth recovering from SUD to reconnect with nature by caring for horses (16). In a study by Madden et al. (2024), when participants from an Indigenous culturally-based SUD program in Northwestern Ontario were asked about time spent on the land, they spoke of their newfound connection to Mother Earth, leading to reflection, clarity, and strength (2).

### 3.3 Barriers for Indigenous persons seeking land-based treatment for SUD

Although successful land-based interventions exist, some barriers prevent Indigenous persons from seeking care. Firstly, if Western medical treatments are incorporated into land-based healing, language barriers may lead to potential misunderstandings between providers and Indigenous persons (11). Language barriers and complex terminology may contribute to power imbalances and mistrust in the provider. Additionally, negative past experiences of discrimination with Westernized healthcare may act as a barrier for those with SUD (17). Biologically, patterns of addiction are the same for Indigenous and non-Indigenous individuals. However, historical and modern-day effects of colonization have led to additional trauma that must be addressed alongside SUD, revealing the importance of a culturally safe environment for healing (14, 17).

A trauma-informed care approach must be taken when supporting Indigenous persons with SUD (18). Countless experiences of historical and current discrimination, including loss of land and culture, residential schools, and missing and murdered Indigenous women, contribute to trauma that makes recovery more complex. Consistently validating trauma and acknowledging its impact on SUD helps to create a strong therapeutic alliance centered around healing (19).

## 4 Discussion

Based on challenges noted throughout the literature, the following recommendations can be made to support future implementation of land-based healing programs for SUD.

### 4.1 Recommendation 1: data sharing on program effectiveness

Although there are reported benefits of land-based SUD treatment, a challenge in future program implementation is the lack of accessible evidence on programs’ effectiveness and outcomes (6, 13). There is often limited description of service structures, financing, and the role of community and healthcare providers. This makes it difficult to receive recognition and funding from the government or donors, and for communities to make informed decisions when initiating their own programs. In the Eabametoong First Nation, an opioid agonist treatment was initiated that was operated within the community, providing cultural and holistic care for SUD (6). Although the community noted the success of the Eabametoong First Nation program, no description of services, funding, or policy was provided, making it difficult for other communities to replicate the program. Likewise, in literature, this makes it difficult to identify causal relationships between key program features and assess the impact on patient outcomes. However, evaluating the success of land-based interventions through a Western lens may not capture the critical contribution of traditional Indigenous knowledge and lived experience (20, 21). Using typical methods of Western data collection, like surveys, may result in poor completion rates due to mistrust by Indigenous persons (21, 22). Historically, there have been countless instances where research conducted in Indigenous communities failed to benefit the community with potentially negative impacts, so there is a legacy of mistrust surrounding academic researchers in many Indigenous communities.

Evaluating land-based SUD program success is difficult due to the complex nature of the programs, which are often associated with unique cultures, geographies, resources, and potential combinations of Westernized therapies (6, 13, 20, 21, 23). Using a typical Western approach to analyze success may not be appropriate as it may not account for Indigenous persons’ experiences and traditional knowledge (24). A scoping review by the *Hotıì ts’eeda and NWT Recreation and Parks Association* found that the successes of land-based programs were poorly documented, which may be because Indigenous knowledge is often traditionally shared through oral language rather than documentation (23). The review examined land-based programs across various sources of publicly available data, like PowerPoints and videos, not only academic literature. It found limitations in land-based healing documentation, supporting reinforcement on the recommendation for effective data sharing.

Going forward, a framework is needed to evaluate land-based programs that includes quantitative and qualitative data, while focusing on traditional Indigenous ways of knowledge translation, such as oral storytelling (23, 24). Effective knowledge describing the efficacy of land-based programs would allow other communities to implement their own land-based treatment centers for SUD. Methods of data collection that consider cultural, spiritual, and social factors will be more appropriate and accepted in demonstrating the effectiveness of land-based treatments, supporting the implementation of future land-based addiction treatment with other communities and within healthcare institutions (6, 13, 14, 20). The importance of this recommendation is supported through assessment of the Naandwe Miikan Clinic, as researchers and staff were unable to access statistical trends from patient records, decreasing objective outcome measurement and informed decision making (6).

### 4.2 Recommendation 2: funding equity

Another challenge in the implementation of land-based treatment for SUD is funding equity. The Naandwe Miikan clinic, a land-based opioid agonist treatment center, recommends a change in how government funds are allocated (6). The clinic emphasizes a Two-Eyed Seeing approach, appreciating collaboration between Western and land-based cultural treatment. However, it was reported that majority funding was allocated to physicians and pharmacists, neglecting those who provide cultural healing, such as Indigenous counsellors. Equitable funding models must be developed for more land-based programs to thrive (6, 25). Elders and Indigenous staff members should receive equitable compensation for their time, rather than the bulk of the funding being distributed among Western health professionals. Josewski et al. (2023), conducted interviews with 33 providers involved in seven urban Indigenous mental health and addiction organizations, who described ongoing funding struggles and the need for growing recognition of Indigenous approaches to care (25). These programs were more culturally-based than land-based, with a focus on mental health, potentially decreasing applicability to this review, but still highlighting struggles in inequitable funding. Otherwise, the Naandwe Miikan Clinic was the sole program to report challenges in funding equity between Indigenous and Western providers (6). Although this recommendation may be strengthened by increased reporting in academic literature, funding concerns may be underreported in the context of Western literature, and may be well-described in Indigenous knowledge translation. This recommendation cannot be disregarded and calls on the need for better funding evaluation in literature.

### 4.3 Recommendation 3: collaboration to provide culturally safe care

Collaboration between Western and Indigenous healthcare providers represents a major challenge in the implementation of culturally safe treatments for SUD. A systematic review by Urbanoski (2017) of 19 interventions for SUD found that the collaborative approach between Indigenous culture-based and Western treatments improves overall well-being and decreases substance use (26). Limitations to this review were self-identified as minimal service descriptions, emphasizing the prior recommendation of increased data sharing with a focus on Indigenous knowledge translation.

Willie Ermine’s concept of Ethical Space perfectly encapsulates the importance of collaboration between Indigenous and Western care providers in the treatment of SUD (27). Ethical space is a theoretical space created when two differing viewpoints engage, such as Indigenous and Western thought. Ethical Space facilitates collaboration and creates a bridge between societies while appreciating the unique diversity of each. When members of the Mushkegowuk First Nation were asked about challenges in offering land-based interventions, the response was that there was a need for collaboration between Western professionals and natural healers (8). Limited by small sample sizes, this study can be used in conjunction with others to strengthen the recommendation for collaboration and cultural safety training.

Suggested next steps include developing regulations that require all care providers to take a cultural safety training course that is co-designed with local Indigenous communities before involvement in Indigenous addictions care, which may promote collaboration between Indigenous and Western healers to ensure holistic land-based healing for SUD (14, 17). The Indigenous Healing and Seeking Safety (IHSS) Approach is a model of culturally safe addiction treatment used in the Benbowopka Treatment Centre, servicing the North Shore of Lake Huron (13, 28). Healthcare providers and traditional healers complete five days of IHSS cultural safety training before program involvement, which uses a Two-Eyed Seeing approach and involves sharing circles with Elders, Grandfather Teachings, smudging, and prayer. This training also allows the providers to learn more about their own identity, self-care, setting boundaries, and coping with anger or PTSD. Not only can training teach Westernized healthcare providers about cultural aspects of health, but it can also teach traditional healers about Western healthcare options available to assist in recovery (17). Patient outcomes before and after IHSS implementation were compared in a pre-implementation group of 266 clients and an intervention group of 136 clients (13, 28). When statistical adjustment accounted for confounding variables in patient populations, such as prior SUD treatment attempts and living out of community, it was found that IHSS training increased program completion rates, with an odds ratio of 1.95 (28). Statistically, there was no impact on access to healthcare services between groups. Overall, this provides strong evidence on the potential benefits of cultural safety training, but further research is required to solidify the correlational relationship between cultural training, collaboration, and land-based SUD program completion.

### 4.4 Recommendation 4: holistic approach

Finally, it is recommended that land-based SUD programs take a holistic healing approach by addressing exercise, eating well, safe living conditions, education, and employment (6). The Naandwe Miikan clinic takes a Minobimaadiziwin approach, which translates to ‘living a good life’ (6). Community services like employment, housing, policing, justice, family services, and social services are incorporated in SUD healing to help the individual thrive in all aspects. Many other land-based healing programs report similar approaches, including Blood Tribe Youth Wellness Centre, Leading Thunderbird Lodge, and Rising Sun (29). Twenty-two qualitative interviews were conducted with participants and providers at the Naandwe Miikan clinic (6). Themes were analyzed using research software and reviewed with available program documentation. Small sample sizes and participant recruitment methods relying on volunteers may not represent the participant population, so further evaluation of the impact of a holistic approach on patient outcomes may strengthen this recommendation.

### 4.5 Limitations

This article is limited by the small research team conducting the literature screening, creating potential for selection bias. Likewise, identification of themes was based on author interpretation, without a statistical method of objective data analysis. This review included programs within Canada, which may decrease the applicability outside of that region. Lastly, program outcomes are often underreported, leading to limited data on program effectiveness. Despite these limitations, this review provides a comprehensive overview of the current state of land-based SUD treatment in Indigenous populations in Canada, with future recommendations.

## 5 Conclusion

In Canada in 2017, over 4000 Indigenous peoples died because of opioids (30). Likewise, 10% of overdose deaths were Indigenous peoples, despite making up less than 3% of the population. Canada must do better. Land-based healing offers benefits for Indigenous persons recovering from SUD, including strengthening relationships with Mother Earth, ancestors, language, identity, community, and family (7, 13). Through increasing awareness on key features and outcomes of land-based SUD treatments, as seen in **Table 1**, more programs can be implemented across Canada to support Indigenous healing (16, 17).

**Table 1:** Summary of land-based programs discussed, along with their key features and reported outcomes.

Program	Reference	Key features	Reported outcomes
Naandwe Miikan Clinic	(6)	Available locally, Two-Eyed Seeing Approach, holistic services	Community-led and culturally-safe services accepted by Indigenous community members. Limitations noted in objective documented outcomes
Nishnawbe Aski First Nation NAN Hope Program	(7)	No referrals are required and community members can reach out directly to receive support	Opioid treatment retention rate after 18 months was 72%
Mushkegowuk First Nation	(8)	Facilitates intergenerational connection and family involvement Programming for mental health and addiction	Subjective reports of healing connections between family, land, culture, language, and self-identity
The Gwekwaadziwin Miikan Mental Health and Addiction Program	(15)	Three-phase program providing continuity of care	No reported outcomes
Nimkee NupiGawagan Healing Centre	(16)	Equine Assisted Learning encourages connection with horses, and therefore to the land	Found to improve spiritual and mental wellness through three main factors: spiritual exchange with the horse, improvement of non-verbal communication, and increased compassion
Eabametoong First Nation	(6)	Community-based, holistic approach to care	Community noted success, but there are no reported outcomes to objectively measure success
Benbowopka Treatment Centre	(13, 28)	Implementation of cultural safety training (Indigenous Healing and Seeking Safety (IHSS))	IHSS associated with higher participant completion rates, but no change to health service usage (primary care and emergency department)

To improve and refine these programs, existing literature demonstrates strong recommendations for increased data sharing on program effectiveness and use of a collaborative Two-Eyed Seeing approach with cultural safety training (6, 8, 13, 14, 17, 20–24, 26, 28). It is important to recognize that calls for “rigor” within review methodology often reflect Western epistemological standards that may not fully account for Indigenous knowledge systems. In the context of land-based healing, cultural integrity, relational accountability, and spiritual alignment are themselves forms of methodological rigor. Our collaborative process with an Indigenous knowledge holder was central to ensuring that this review reflects traditional teachings and the lived realities of communities engaging in land-based wellness practices.
